# Reconstructing the Migratory Behavior and Long-Term Survivorship of Juvenile Chinook Salmon under Contrasting Hydrologic Regimes

**DOI:** 10.1371/journal.pone.0122380

**Published:** 2015-05-20

**Authors:** Anna M. Sturrock, J. D. Wikert, Timothy Heyne, Carl Mesick, Alan E. Hubbard, Travis M. Hinkelman, Peter K. Weber, George E. Whitman, Justin J. Glessner, Rachel C. Johnson

**Affiliations:** 1 Institute of Marine Sciences, University of California Santa Cruz, Santa Cruz, California, United States of America; 2 US Fish and Wildlife Service, Lodi, California, United States of America; 3 Tuolumne River Restoration Center, California Department of Fish and Wildlife, La Grange, California, United States of America; 4 School of Public Health, Division of Biostatistics, University of California, Berkeley, California, United States of America; 5 Cramer Fish Sciences, Auburn, California, United States of America; 6 Glenn T. Seaborg Institute, Lawrence Livermore National Laboratory, Livermore, California, United States of America; 7 Department of Animal Sciences, University of California Davis, Davis, California, United States of America; 8 UC Davis Interdisciplinary Center for Plasma Mass Spectrometry, Department of Geology, University of California Davis, Davis, California, United States of America; 9 National Marine Fisheries Service, Southwest Fisheries Science Center, Santa Cruz, California, United States of America; Pacific Northwest National Laboratory, UNITED STATES

## Abstract

The loss of genetic and life history diversity has been documented across many taxonomic groups, and is considered a leading cause of increased extinction risk. Juvenile salmon leave their natal rivers at different sizes, ages and times of the year, and it is thought that this life history variation contributes to their population sustainability, and is thus central to many recovery efforts. However, in order to preserve and restore diversity in life history traits, it is necessary to first understand how environmental factors affect their expression and success. We used otolith ^87^Sr/^86^Sr in adult Chinook salmon (*Oncorhynchus tshawytcha*) returning to the Stanislaus River in the California Central Valley (USA) to reconstruct the sizes at which they outmigrated as juveniles in a wetter (2000) and drier (2003) year. We compared rotary screw trap-derived estimates of outmigrant timing, abundance and size with those reconstructed in the adults from the same cohort. This allowed us to estimate the relative survival and contribution of migratory phenotypes (fry, parr, smolts) to the adult spawning population under different flow regimes. Juvenile abundance and outmigration behavior varied with hydroclimatic regime, while downstream survival appeared to be driven by size- and time-selective mortality. Although fry survival is generally assumed to be negligible in this system, >20% of the adult spawners from outmigration year 2000 had outmigrated as fry. In both years, all three phenotypes contributed to the spawning population, however their relative proportions differed, reflecting greater fry contributions in the wetter year (23% vs. 10%) and greater smolt contributions in the drier year (13% vs. 44%). These data demonstrate that the expression and success of migratory phenotypes vary with hydrologic regime, emphasizing the importance of maintaining diversity in a changing climate.

## Introduction

Life history diversity is often cited as a crucial component of population resilience, based on theoretical and empirical evidence that asynchrony in local population dynamics reduces long-term variance and extinction risk at both regional and metapopulation scales [1]. Pacific salmon are recognized for their complex life histories, having evolved alongside the shifting topography of the Pacific Rim [2]. In the California Central Valley (CCV), four runs of imperilled Chinook salmon (*Oncorhynchus tshawytscha*) coexist, exhibiting asynchronous spatial and temporal distributions that allow them to exploit a range of ecological niches [3,4]. The maintenance of multiple and diverse salmon stocks that fluctuate independently of each other has been shown to convey a stabilizing ‘portfolio effect’ to the overall the stock-complex [5,6]. Such ‘risk spreading’ can also act at finer scales [7,8], such as within-population variation in the timing of juvenile emigration. Preserving and restoring life history diversity remains an integral goal of many salmonid conservation programs [9], yet baseline monitoring data with which to detect and respond to changes in trait expression are scarce and difficult to relate directly to population abundance.

**Fig 1:**
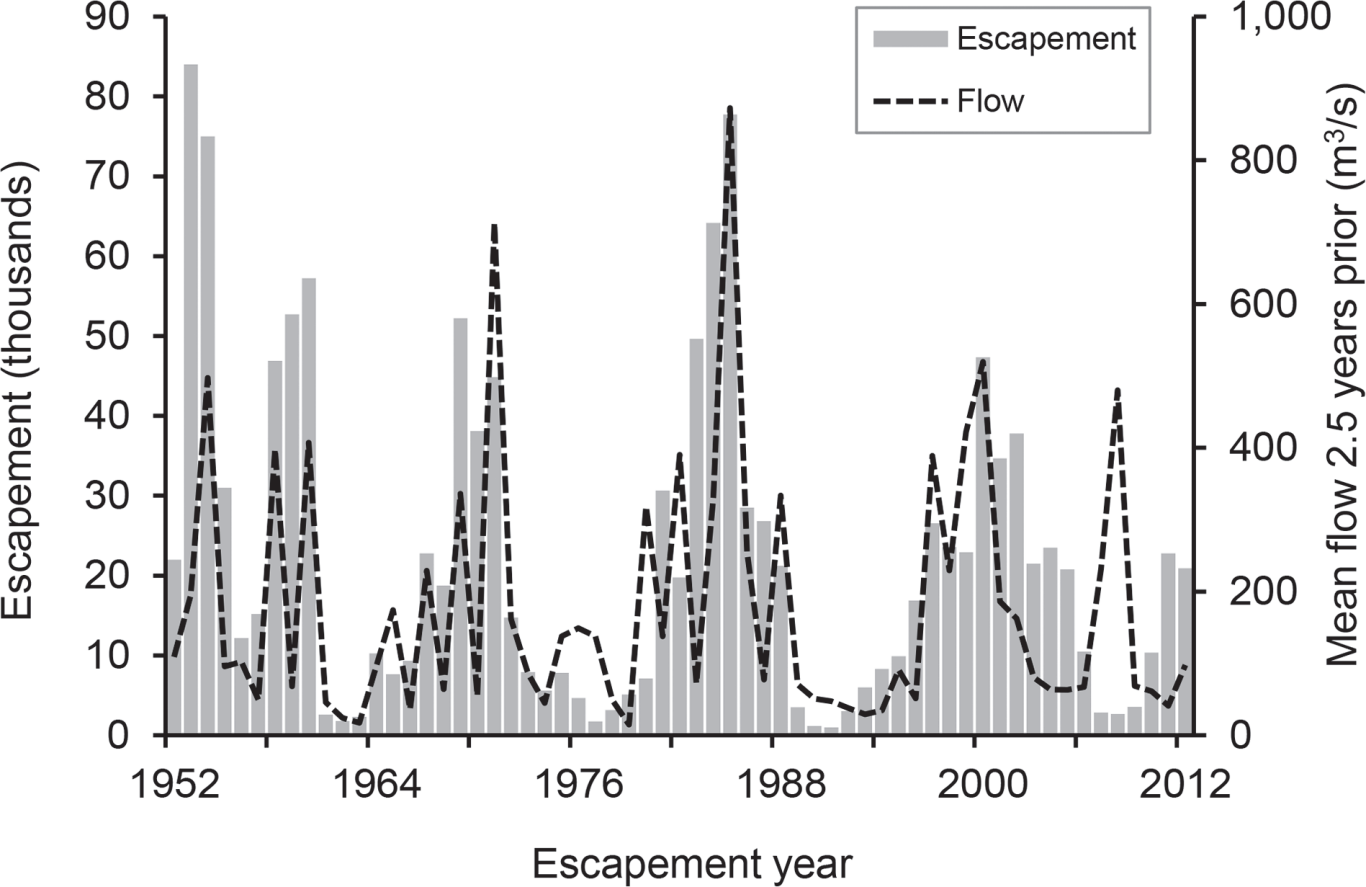
Relationship between adult salmon returns to the San Joaquin basin and the river flows experienced as juveniles. Fall-run Chinook salmon returns (‘escapement’) to the San Joaquin basin from 1952 to 2011 (CDFW GrandTab, www.CalFish.org) relative to mean flows at Vernalis (USGS gauge 11303500, http://waterdata.usgs.gov/nwis) for the January to June outmigration period they experienced 2.5 years previous. Note that adult abundance estimates have not been corrected for age distributions (we assumed that all adults returned at age 3), inter-annual variation in harvest rates or out-of-basin straying. The large deviation in 2007 reflected poor returns that were attributed to poor ocean conditions [96] and resulted in the closure of the fishery. Adapted from [97].

The expression and success of certain traits can be largely driven by hydroclimatic conditions experienced during critical periods of development [10]. CCV Chinook salmon are at the southern margin of their species range, and are subjected to highly variable patterns in precipitation and ocean conditions [4,11]. It is also a highly modified system, with >70% of spawning habitat lost or degraded as a result of mining activities, dam construction, and water diversions [4,12]. The majority of salmon rivers in the CCV experience regulated flows according to ‘water year type’ (WYT). Optimization of reservoir releases presents considerable challenges, given often limited availability and multiple uses of the water resource, inability to predict annual precipitation, and uncertainty surrounding the direct and indirect effects of flow on salmon survival [13]. Such challenges are particularly critical for the more southerly San Joaquin basin, whose salmon populations fluctuate considerably with river flows experienced during juvenile rearing (Fig 1).

Juvenile Chinook salmon exhibit significant variation in the size, timing and age at which they outmigrate from their natal rivers [3,14]. Selection for one strategy over another may vary as a function of freshwater and/or marine conditions [10,15]. In the CCV, fall-run juveniles typically rear in freshwater for one to four months before smoltification prompts downstream migration toward the ocean [16]. In this system, contributions of the smaller fry and parr outmigrants to the adult population are often assumed to be negligible, as survival tends to correlate with body size [17,18] and there is little evidence for downstream rearing in the San Francisco estuary [19]. However, this has never been explicitly tested for smaller size classes. Indeed, salmon fry are frequently observed rearing in tidal marsh and estuarine habitats in other systems [3], and have been observed in non-natal habitats in the CCV, such as the mainstem Sacramento and San Joaquin Rivers, freshwater delta, and estuary [20]. Juvenile salmon that enter the ocean at a larger size and have faster freshwater growth have demonstrated a survival advantage when faced with poor ocean conditions [18]. Yet intermediate size classes can be better represented in the adult population [21,22], and size-selective mortality can be moderated by a variety of other processes [23]. In a regulated system such as the CCV, identifying the relationships between observable traits, hydroclimatic regime and survival would be invaluable for reducing uncertainty and predicting how populations may respond to climate change and management actions related to water operations.

Quantifying the relative contribution of fry, parr and smolt outmigrants to the adult population has, until now, been largely limited by the methodological challenges associated with reconstructing early life history movements of the adults. Mark-recapture studies using acoustic and coded wire tags (CWT) have provided empirical indices of juvenile survival through stretches of the Sacramento-San Joaquin River Delta (hereafter, “the Delta”) [24,25], but are hindered by low rates of return and tend to utilize hatchery fish that may exhibit different rearing behavior and sea-readiness to their wild counterparts [26]. Furthermore, ‘fry pulses’ tend to be dominated by individuals <45mm FL, which are difficult to mark externally without causing damage or behavioral modifications. No study to date has tracked habitat use of individual salmon over an entire lifecycle to estimate the relative success of juvenile outmigration phenotypes under different flow conditions. Previous studies have tended to rely on correlations between environmental conditions (e.g. flow) experienced during outmigration and the abundance of returns (Fig 1) [27]. Recent advances in techniques using chemical markers recorded in biomineralised tissues provide rare opportunity to retrospectively “geolocate” individual fish in time and space [28]. Given their incremental growth and metabolically inert nature, otoliths (‘ear stones’) represent a unique natural tag for reconstructing movement patterns of individual fish [29]. The technique relies on differences in the physicochemical environment producing distinct and reproducible “fingerprints” in the otolith. In the CCV, strontium isotopes (^87^Sr/^86^Sr) are ideal markers because the water composition varies among many of the rivers and is faithfully recorded in the otoliths of Chinook salmon [30–32]. Changes in otolith ^87^Sr/^86^Sr values can be used to reconstruct time- and age-resolved movements as salmon migrate through the freshwater and estuarine environments [33]. Furthermore, otolith size is significantly related to body size [34,35], allowing back-calculation of individual fork length (FL) at specific life history events [36].

Here, we document metrics of juvenile life history diversity (phenology, size, and abundance) of fall-run Chinook salmon as they outmigrated from the Stanislaus River during an ‘above normal’ (2000) and ‘below normal’ (2003) WYT. We used otolith ^87^Sr/^86^Sr and radius measurements to reconstruct the size at which returning (i.e. “successful”) adults from the same cohort had outmigrated, then combined juvenile and adult datasets to estimate the relative contribution and survival of fry, parr and smolt outmigrants. Our main objectives were to determine (1) if a particular phenotype contributed disproportionately to the adult spawning population, (2) whether this could be attributed to selective mortality, and (3) if patterns in phenotype expression and success varied under contrasting flow regimes.

## Study Area

**Fig 2:**
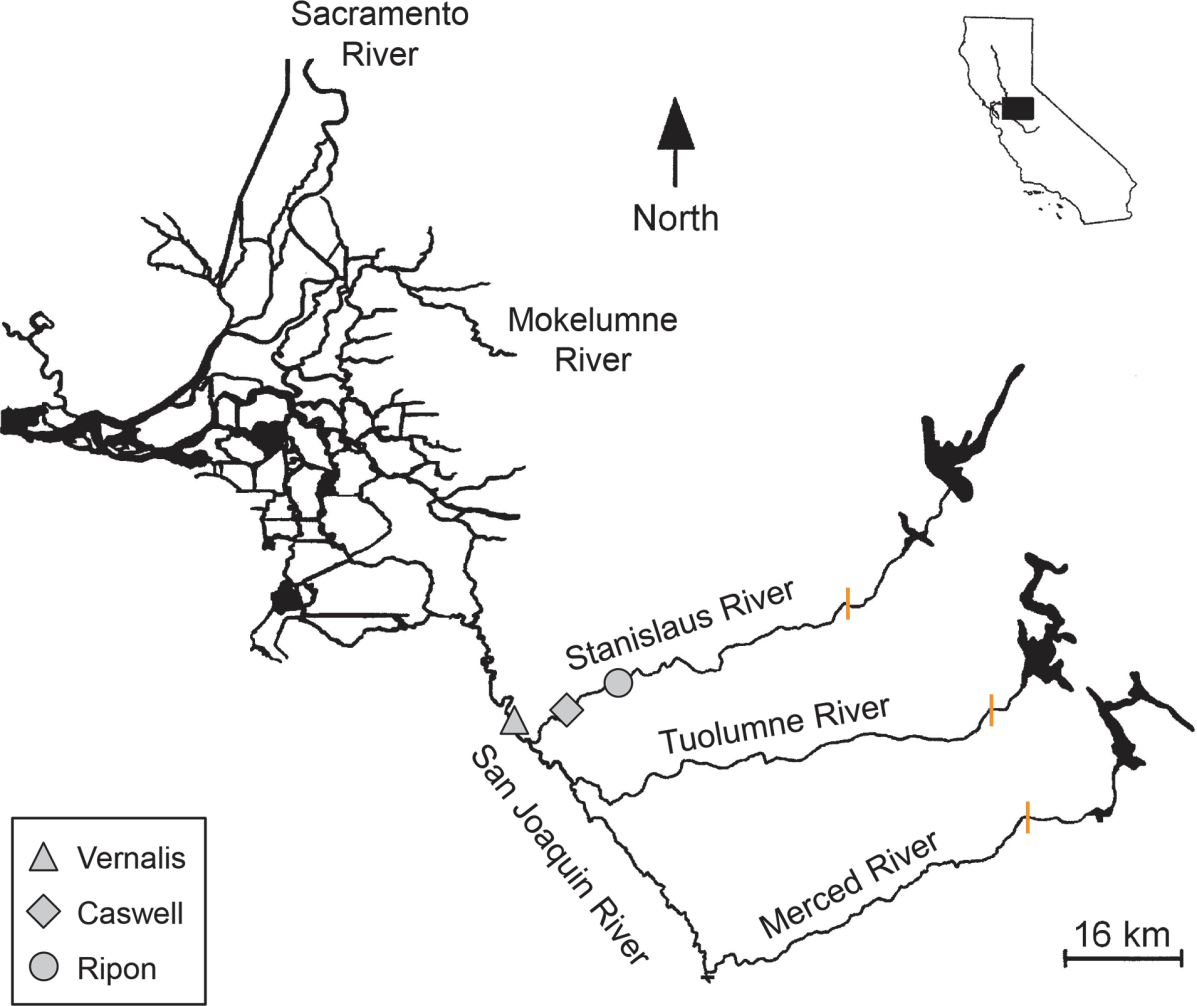
The San Joaquin basin of the Central Valley, California (inset). Map showing the major rivers in the San Joaquin basin, and the location of the rotary screw trap site at Caswell Memorial State Park and USGS gauges at Ripon and Vernalis. The upstream barriers to salmon migration in the three main tributaries are indicated by orange bars.

The Stanislaus River (hereafter, “the Stanislaus”) is the northernmost tributary of the San Joaquin River, draining 4,627 km^3^ on the western slope of the Sierra Nevada (Fig 2) [37]. The basin has a Mediterranean climate and receives the majority of its annual rainfall between November and April. Contrasting with the Sacramento watershed in the north, the hydrology of the San Joaquin basin is primarily snowmelt driven [4]. There are over 40 dams in the Stanislaus, which collectively have a capacity of 240% of the average annual runoff [38]. Historically, the Stanislaus contained periodically-inundated floodplain habitat and supported spring- and fall-run Chinook salmon; however, spring-run salmon were extirpated by mining and dam construction, reducing habitat quality and preventing passage to higher elevation spawning grounds [4].

## Materials and Methods

### Ethics statement

This research was conducted in strict accordance with protocols evaluated and approved by the University of California, Santa Cruz Institutional Animal Care and Use Committee for this specific study (permit number BARNR1409). Otolith and scale samples were collected by California Department of Fish and Wildlife (CDFW) staff from adult salmon carcasses (i.e. already expired) as part of their annual carcass survey, permitted under the State legislative mandate to perform routine management actions. No tissue collections were taken from any state- or federally-listed endangered or protected species for this study.

### Juvenile sampling and hydrologic regime

Typically, fall-run Chinook salmon return to the San Joaquin basin from September to early January, and their offspring outmigrate the following January to June [16,39]. Juveniles were sampled as they left the Stanislaus using rotary screw traps (RST) at Caswell Memorial State Park (Fig 2, N 37°42'7.533", W 121°10'44.882). Sampling was terminated when no juveniles had been captured for at least seven consecutive days in June or July [40]. Here, we focused on an ‘above normal’ (2000) and ‘below normal’ (2003) WYT, and defined the outmigration period as January 1 to June 30, inclusive. When traps were checked, all fish were counted and up to 50 were randomly selected for fork length (FL) and weight measurements. Given potential subjectivity in visual staging criteria [41], we defined migratory phenotypes (fry, parr and smolt) by size: ≤55mm, >55 to ≤75mm, and >75mm FL, respectively (after [21]). Unmeasured fish were assigned to phenotype using the observed proportions in the measured fish for the same date. For each phenotype, we interpolated missing catch values with a triangular weighted mean [42].

Marked fish were periodically released to develop a statistical model of trap efficiency, which was used to expand counts of fry, parr and smolt-sized outmigrants. Trap efficiency was estimated using a GLM with a quasibinomial error distribution because of overdispersion in capture probabilities. We used the same efficiency model as [42], only using phenotype (fry, parr, smolt) to characterize fish size, rather than FL. We propagated uncertainty by deriving estimated expanded counts from repeated Monte Carlo draws (n = 2000) from the estimated sampling distribution of the estimated coefficients from the logistic efficiency model using R package mvtnorm [43]. Daily flow observations (USGS gauge no. 11303000 at Ripon, www.waterdata.usgs.gov/nwis) were used with the randomly-sampled model coefficients to simulate daily trap efficiency. Passage estimates were then simulated using daily catch and simulated trap efficiencies. We incorporated extra-binomial variation by generating simulated daily catch values from a beta-binomial distribution (based on the simulated efficiencies and passage estimates, as well as the dispersion estimated from the efficiency model). Finally, new daily passage estimates were calculated using simulated catch and trap efficiencies. Thus the final passage estimates incorporate both sampling error (catch) and estimation error (efficiency model). Annual passages estimates and confidence intervals (2.5% and 97.5% quantiles) were generated by summing daily passage estimates for the 6 month outmigration period (i.e. n = 2000 x 180 days).

Measured daily size-frequency distributions were applied directly to the expanded abundance estimates, then grouped into 2mm FL bins. We attempted to produce passage estimates by FL, but the distribution used in the uncertainty propagation procedure (see above) is asymmetric at low catches, resulting in zero-inflation and the median of the resampled distribution often being lower than the observed raw catch.

Turbidity was measured at Caswell using a LaMott turbidity meter [40]; mean daily flow and maximum daily temperature were measured at Ripon (gauge details above). Daily passage estimates, turbidity, flow and temperature were log_10_ transformed, then averaged for the 6-month outmigration period and compared among years by ANOVA, adjusting for temporal autocorrelation using the Durbin-Watson (DW) test [44]. Pearson's chi-squared test was used to identify differences in the proportion of phenotypes among years. Fry, parr and smolt phenology was summarized using three metrics associated with their date of passage past the trap: the range, interquartile range (IQR), and median (or “peak”) outmigration date. Phenotype “migratory periods” were defined as the maximum IQR for both years combined.

### Adult sampling and cohort reconstruction

To track outmigration cohorts 2000 and 2003 into the adult escapement, sagittal otoliths were extracted from Chinook salmon carcasses (aged 2–4 years, 45–112 cm FL) collected in the 2001–2006 CDFW Carcass Surveys (Table 1). Unmarked fish were sampled randomly, but in earlier years, known-hatchery fish with CWTs and clipped adipose fins (“adclipped”) were preferentially sampled to assess the accuracy of age estimations. We utilized all otoliths collected from all unmarked fish, but included a subset of CWT fish from outmigration year 2000 (n = 27), which we analyzed blind to assess the accuracy of our natal assignments. Ages were estimated by counting scale annuli [45,46]. Each scale was aged by at least two independent readers and discrepancies resolved by additional reading(s).

**Table 1:** Adult sample sizes, age structure and collection periods.

	Outmigration cohort 2000 (wetter)			Outmigration cohort 2003 (drier)		
Age	N	%	Collection period	N	%	Collection period
2	6	7%	11/20/01–12/06/01	2	2%	11/08/04–11/12/04
3	80	87%	10/07/02–12/12/02	56	67%	11/02/05–12/15/05
4	6	7%	11/12/03–12/04/03	25	30%	11/15/06–12/06/06

Otoliths were analyzed from salmon carcasses belonging to adults that had outmigrated in 2000 and 2003, including 27 known-origin fish included as a blind test of our natal assignments.

### Otolith ^87^Sr/^86^Sr analyses

Otolith strontium isotope ratios (^87^Sr/^86^Sr) were measured along a standardized 90° transect [47] by multiple collection laser ablation inductively coupled plasma mass spectrometry (MC-LA-ICPMS; Nu plasma HR interfaced with a New Wave Research Nd:YAG 213 nm laser). Spot analyses were used to allow coupling of chemical data with discrete microstructural features, but otherwise preparation and analysis methods followed those of Barnett-Johnson et al. [32,48]. In brief, otoliths were rinsed 2–3 times with deionized water and cleaned of adhering tissue. Once dry, otoliths were mounted in Crystalbond resin and polished (600 grit, 1500 grit then 3 μm lapping film) until the primordia were exposed. Depending on sample thickness and instrument sensitivity, a 40–55μm laser beam diameter was used with a pulse rate of 10-20Hz, 3–7 J/cm^2^ fluence, and a dwell time of 25–35 seconds, resulting in individual ablations roughly equivalent to 10–14 days of growth. Where individual ablations exhibited isotopic changes with depth (e.g. at habitat transition zones), only the start of the ablation was used (e.g. S1 Fig). Helium was used as the laser cell carrier gas (0.7–1.0 L/min) to improve sample transmission and was mixed with argon before reaching the plasma source. Krypton interference (^86^Kr) was blank-subtracted by measuring background voltages for 30 s prior to each batch of analyses, and ^87^Rb interferences were removed by monitoring ^85^Rb. Isotope voltages were integrated over 0.2 s intervals then aggregated into 1 s blocks. Outliers (>2SD) were rejected. Marine carbonate standards (‘UCD Vermeij Mollusk' and *O*. *tshawytscha* otoliths) were analyzed periodically to monitor instrument bias and drift, producing a mean mass-bias corrected ^87^Sr/^86^Sr ratio (normalized to ^86^Sr/^88^Sr = 0.1194) within 1SD of the global marine value of 0.70918 (0.70922 ± 0.00008 2SD).

### Strontium isotopes to reconstruct natal origin and size at outmigration

The baseline of natal ^87^Sr/^86^Sr signatures described in [32] was updated and expanded upon to increase sample sizes and among-year representation, resulting in an ‘isoscape’ that encompassed all major CCV sources, with many sampled across multiple years and hydrologic regimes. Linear discriminant function analysis (LDFA) was used to predict the natal origin of the sampled adult spawners, assuming equal prior probabilities for all sites (S1 Text). Differences in natal ^87^Sr/^86^Sr values were tested between years and sites (S1 Text, S1 Table and S2 Fig), and the performance of the LDFA was assessed using known-origin reference samples (S2 Table). Adults in this study were considered strays (not produced in the Stanislaus) when their natal ^87^Sr/^86^Sr were closer to other sources in the isoscape, and were excluded from further analysis.

**Fig 3:**
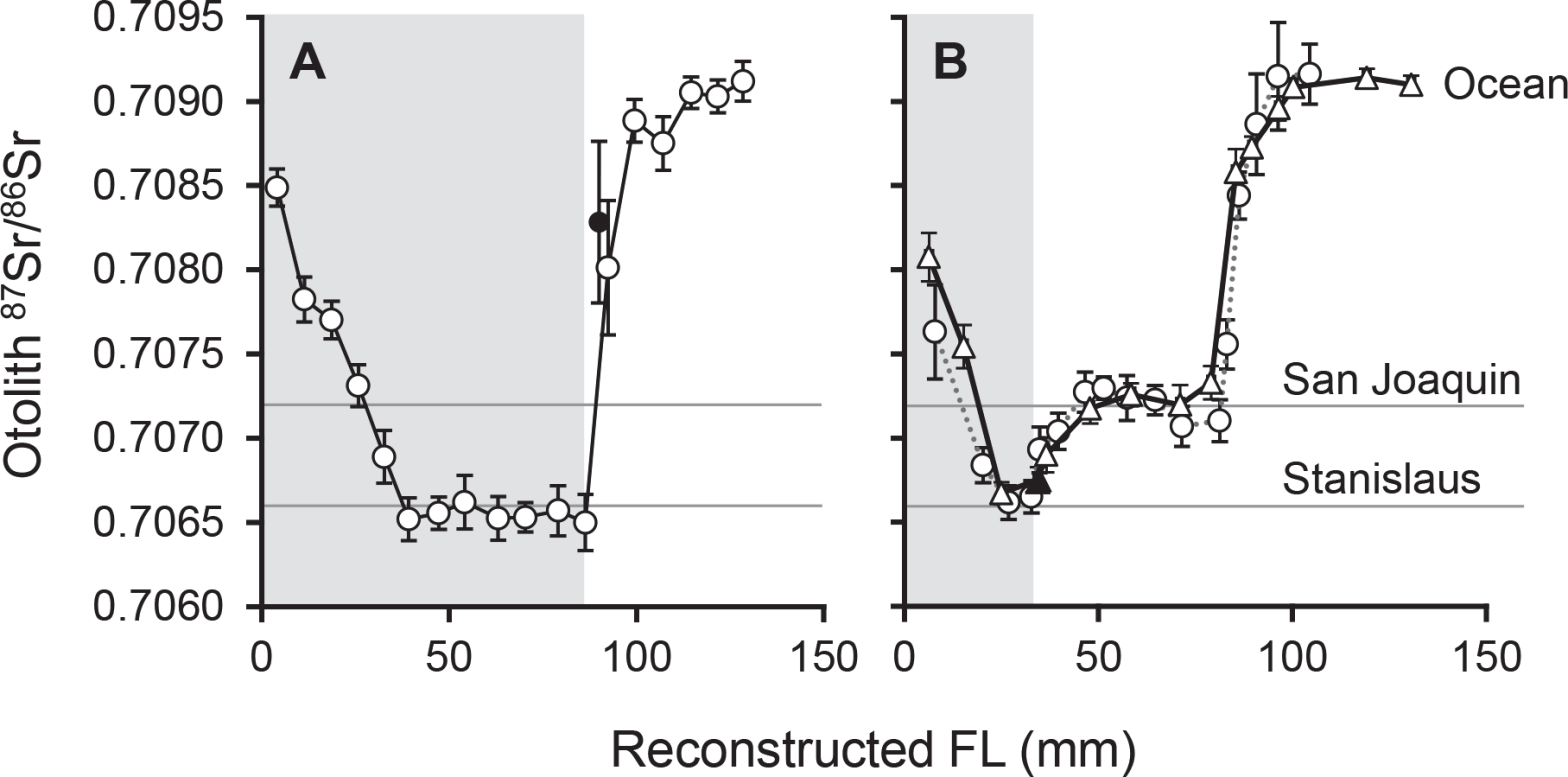
Otolith ^87^Sr/^86^Sr reconstructions of a smolt and fry outmigrant. Otolith ^87^Sr/^86^Sr profiles against back-calculated FL for two adult Chinook salmon that returned to the Stanislaus River having outmigrated as (A) a smolt and (B) a fry. The shaded box indicates the time spent rearing in the natal river. The fry outmigrant reared for several weeks downstream in the San Joaquin River before migrating out to the ocean, as indicated by both the left (triangles, solid line) and right (circles, dashed line) otolith (back-calculated FL = 33.3mm *vs*. 34.9mm). Mean ^87^Sr/^86^Sr signatures for the Stanislaus and San Joaquin Rivers, and modern-day ocean are displayed. Black filled symbols indicate ‘re-spots’ carried out to improve sampling resolution. Error bars = 2SE.

For adults that had successfully returned to the Stanislaus, we monitored the change in ^87^Sr/^86^Sr across the otolith to identify the point at which they had outmigrated as juveniles. The Stanislaus has a significantly lower isotopic value (0.70660 ± 0.00008 SD) than the mainstem San Joaquin River immediately downstream from it (0.70716 ± 0.00013 SD), resulting in a clear increase and inflection point in otolith ^87^Sr/^86^Sr at natal exit (e.g. Fig 3B). If the inflection point was unclear, sequential spot analyses were analyzed by LDFA, and exit was defined as a >0.3 decrease in posterior probability of Stanislaus-assignment to a probability <0.5. Deviation from the mean ^87^Sr/^86^Sr Stanislaus value was assumed to reflect considerable time spent in non-natal water, as (1) the Stanislaus ^87^Sr/^86^Sr signature shows minor variation in otoliths (S1 Table) and water samples collected immediately upstream of the confluence, (2) the RST location is 13.8rkm upstream of the confluence (Fig 2) and (3) the length of time integrated by each laser spot is ~12 days. Therefore, the distance used to back-calculate exit size was from the otolith core to the last natal spot. To improve resolution and accuracy, additional ablations were performed around the transition zone, typically resulting in sub-weekly resolution.

### Reconstructed size at outmigration in the returning adults

The relationship between otolith radius (OR) and FL was first calibrated using juveniles collected from multiple sites in the CCV (S3 Table). All individuals belonged to the same Evolutionarily Significant Unit, which is critical for producing unbiased back-calculation models [49]. As there was no difference in the OR of paired otoliths from single individuals (n = 30, x̄△ = 2.5μm, 95% CI = -5.6–10.6μm), left and right otoliths were used interchangeably. OR was measured along the same 90° transect used for isotope analyses, using a Leica DM1000 microscope and Image Pro Plus (7.0.1).

Reconstructed sizes were grouped into 2mm FL bins and categorized as fry, parr or smolt outmigrants based on the criteria of [21]. Size-frequency distributions were compared between the juvenile and adult samples to identify trends indicative of size-selective mortality. The error around the OR-FL calibration line was used to estimate 95% CI around the proportions of fry, parr and smolt outmigrants using random resampling (n = 5000) of the residuals. This allowed us to derive the relative contribution of each phenotype to the adult spawning population.

### Survival of juvenile migratory phenotypes

To generate survival indices, we normalized the contribution of each phenotype to the adult population by their abundance within each outmigration cohort based on RST sampling. To estimate spawner abundance (“natural escapement”), we removed adclipped strays from total escapement estimates (GrandTab, available at www.calfish.org) using river- and year-specific tag recovery rates (S4 Table), then separated cohorts using annual age distributions [50] and removed unmarked strays using our otolith natal assignments (see results and S4 Table). We evaluated the use of spawner abundance *vs*. “adult production” (after [51]). While production accounts for different harvest rates among years [52], the two metrics produced similar trends in survival (r^2^ = 0.98), and we found that escapement, which includes harvest, bycatch and natural mortality between outmigration and spawning, to be more intuitive to interpret.

The otolith-derived proportions (±95% CI) of phenotype *i* in the escapement (*β*_*i*_) were applied to our natural escapement estimates (*E*_*n*_) to estimate the number of fry, parr and smolt spawners (*E*_*i*_), then *E*_*i*_ was compared with the number of outmigrants of phenotype *i* (*J*_*i*_) to estimate their relative survival (*S*_*i*_):

$$E_{i}=E_{n}\beta_{i}\;S_{i}=E_{i}/J_{i}$$

To estimate 95% CI for *S*_*i*_ we combined error in *β*_*i*_ and *J*_*i*_ using the delta method. The 95% CI for *S*_*i*_ depends on the estimate and its standard error (SE): ${\hat{S}}_{i},\;SE\left({\hat{S}}_{i}\right)$. Assuming independence of *β*_*i*_ and *J*_*i*_, we estimated variance as $SE(\log({\hat{S}}_{i}))≅\sqrt{{\left(\frac{1}{{\hat{J}}_{i}}\right)}^{2}SE^{2}({\hat{J}}_{i})+{\left(\frac{1}{{\hat{\beta }}_{i}}\right)}^{2}SE^{2}({\hat{\beta }}_{i})}$. From this, we derived 95% CI for *S*_*i*_ as $\left(e^{\text{log}({\hat{S}}_{i})-1.96\times SE(\text{log}({\hat{S}}_{i}))},e^{\text{log}({\hat{S}}_{i})+1.96\times SE(\text{log}({\hat{S}}_{i}))}\right)$. Note that uncertainties in adult escapement were not incorporated into these confidence intervals; however, the RST-expansions used to estimate *J*_*i*_ were deemed likely to introduce the largest amount of error.

## Results

### Juvenile outmigration relative to hydrologic regime

**Fig 4:**
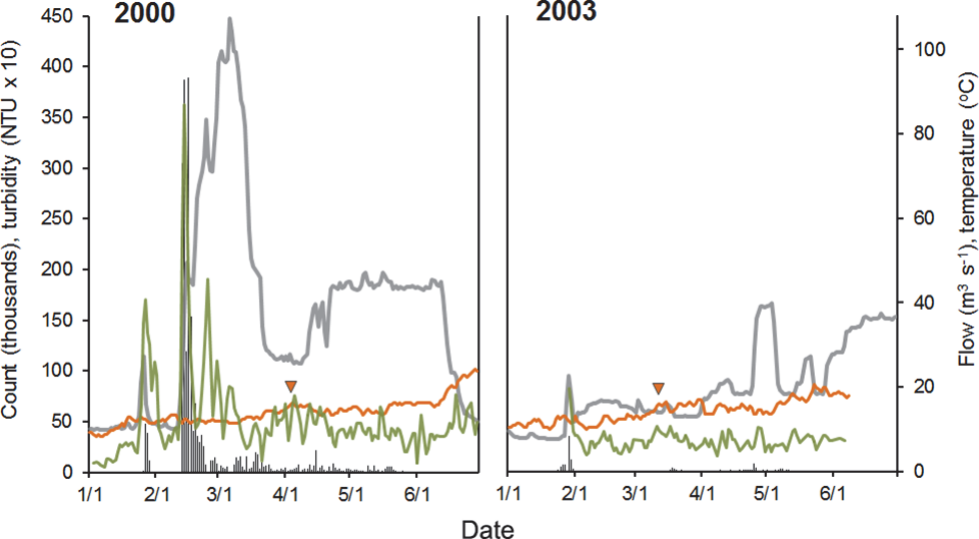
Daily abundance of juvenile salmon outmigrating in 2000 and 2003 relative to ambient environmental conditions. Juvenile salmon were sampled by rotary screw traps at Caswell as they outmigrated from the Stanislaus, and raw counts were expanded into daily abundance estimates (vertical bars) based on trap efficiency models. River flow (grey line) and maximum daily temperature (orange line) were measured at Ripon (data available at http://cdec.water.ca.gov/). Turbidity (green line) was measured at Caswell [40]. The first instance of temperatures reaching 15°C is indicated by an arrow on each plot.

Mean flow and turbidity for the 6 month outmigration period were higher in 2000 than 2003 (DW-adjusted F_1, 361_ = 7.52, p = 0.006 and F_1, 257_ = 14.53, p = 0.0002, respectively) (Fig 4). In the drier year (2003) the river was warmer during the smolt migratory period (Apr 15-May 18: DW-adjusted F_1, 60_ = 4.54, p = 0.037) and peak daily temperatures first exceeded 15°C three weeks earlier (Fig 4).

Peak flows were about five times higher in 2000 than 2003, and accompanied by spikes in turbidity and juvenile migration (Fig 4). The number of outmigrants was an order of magnitude higher in 2000 (Table 2), reflecting significantly higher daily abundances of fry, parr and smolt outmigrants (DW adjusted F_1, 161_ = 11.23, p < 0.001; F_1, 196_ = 47.99, p < 0.001; F_1, 199_ = 6.45, p = 0.0118, respectively). While fry dominated in both years, phenotype contributions differed significantly between years (*X* ^2^ = 223,683, p < 0.001), with parr approximately twice as abundant as smolts in 2000, but vice versa in 2003 (Table 2). One yearling (FL = 140mm) was captured in the RST in 2000, but none in 2003, otherwise the size range of outmigrants was similar between years (25-115mm in 2000 *vs*. 27-115mm in 2003).

**Table 2:** Abundance and migration timing of juvenile migratory phenotypes.

Outmigration cohort	Migratory phenotype	N (95% CI)	Proportion of the sample	Duration of migratory period (range)	Duration of “peak” migratory period (interquartile range)	Peak migration date (median)
2000 (wetter)	Fry	1,837,656 (1,337,351–2,495,523)	0.85	115 d (Jan 2-Apr 25)	4 d (Feb 14-Feb 17)	Feb 16
	Parr	212,042 (141,238–310,174)	0.10	116 d (Feb 4-May 29)	29 d (Mar 18-Apr 15)	Apr 1
	Smolt	101,467 (70,181–145,793)	0.05	110 d (Mar 8-Jun 25)	34 d (Apr 15-May 18)	May 9
	TOTAL	2,151,165 (1,577,638–2,911,393)				
2003 (drier)	Fry	79,862 (59,795–103,916)	0.50	80 d (Jan 23-Apr 12)	4 d (Jan 27-Jan 30)	Jan 29
	Parr	25,729 (17,889–36,282)	0.16	118 d (Feb 5-June 2)	27 d (Mar 18-Apr 13)	Mar 21
	Smolt	55,465 (38,415–76,289)	0.34	107 (Feb 24-Jun 10)	21 d (Apr 18-May 8)	Apr 25
	TOTAL	161,056 (119,868–209,151)				

The abundance and proportions of fry, parr and smolt outmigrants sampled by rotary screw traps, and the timing of their outmigration from the Stanislaus River in 2000 and 2003.

**Fig 5:**
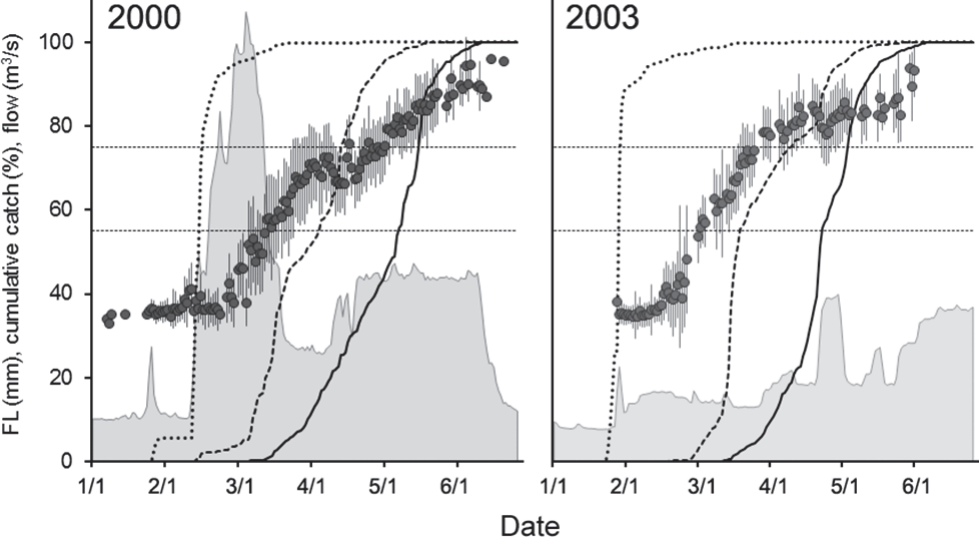
Size and phenology of juveniles outmigrants relative to river flow in 2000 and 2003. Mean (±SD) daily fork length (FL) of juvenile outmigrants, and cumulative percentage of fry (short dashed line), parr (long dashed line) and smolt (solid line) outmigrants relative to flow (filled area). Reference lines indicate the size categories used to define the migratory phenotypes: fry (≤55mm), parr (55-75mm) and smolts (>75mm).

Phenology varied between phenotypes and years (Table 2 and Fig 5). In general, migratory windows were shorter and earlier in the drier year, with smolt outmigration ceasing 15 days earlier in 2003 than in 2000. The peak migratory periods were similar across years for fry and parr, the former exhibiting a compressed interquartile range (4 d) that was tightly correlated with the start of winter flow pulses (Fig 5).

### Natal origin of unmarked adults

The unmarked adults from outmigration cohorts 2000 and 2003 comprised 18% and 51% hatchery strays, respectively, primarily from the Mokelumne, Merced, and Feather River Hatcheries (Table 3). These individuals were removed from subsequent analyses, ensuring that size back-calculations were calculated only for Stanislaus-origin fish that had experienced the same outmigration conditions as the RST-sampled juveniles.

**Table 3:** Natal assignments of unmarked adults based on otolith ^87^Sr/^86^Sr.

Natal source	Outmigration cohort 2000 (%)	Outmigration cohort 2003 (%)
Stanislaus River	82	49
Mokelumne River Hatchery	11	39
Merced River Hatchery	2	1
Feather River Hatchery	5	7
Nimbus Hatchery	2	2
Thermalito Rearing Annex ^a^		1

Natal assignments of unmarked adults fish captured in the Stanislaus River between 2001 and 2006 that outmigrated in 2000 and 2003.

^a^ Part of the Feather River Hatchery

### Back-calculation of size at outmigration

**Fig 6:**
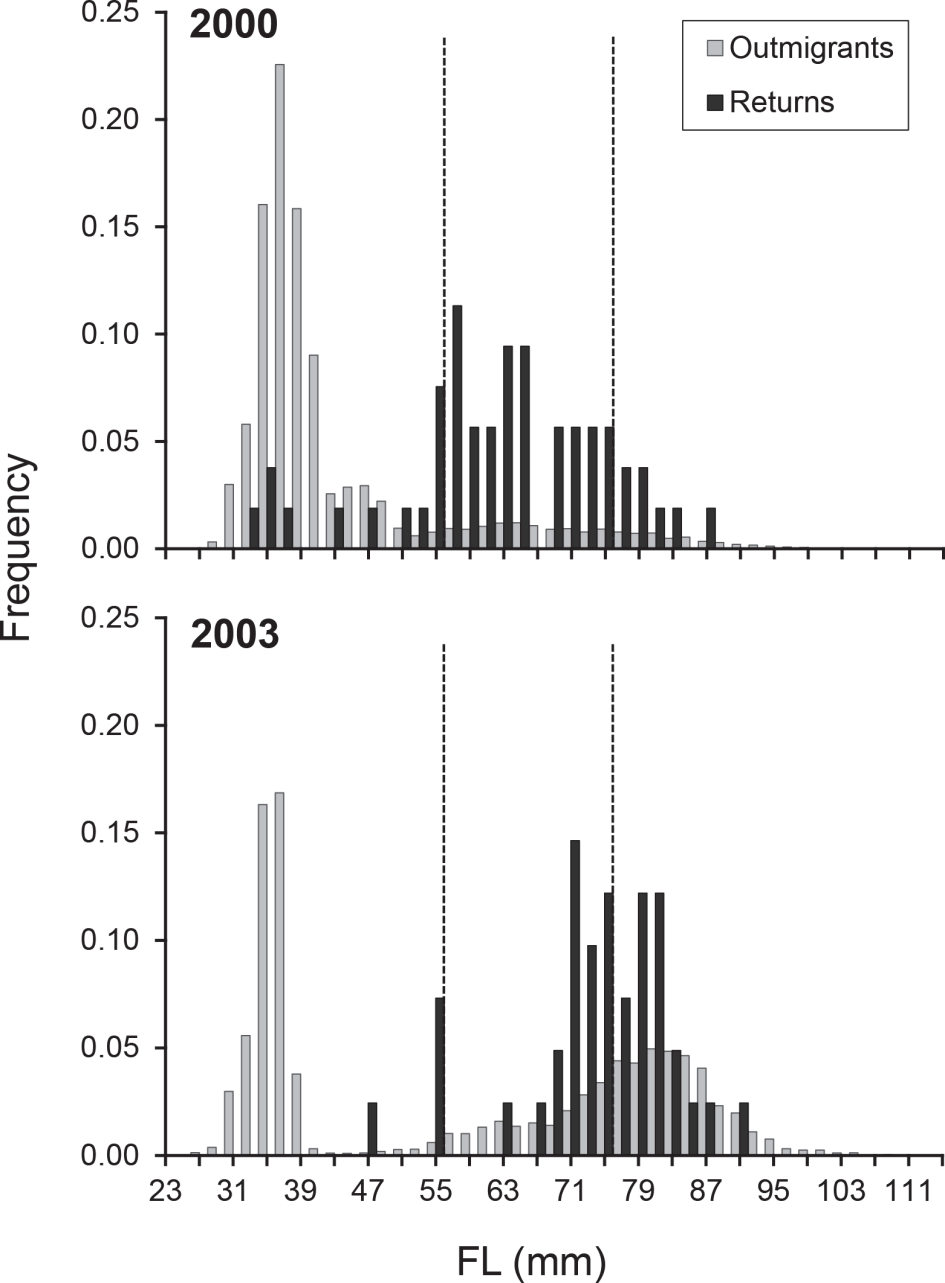
Size-at-outmigration of the juveniles and surviving adults that left freshwater in 2000 and 2003. Size-frequency distributions showing the fork length (FL) at which juveniles outmigrated from the Stanislaus River in 2000 and 2003 (grey bars) and the reconstructed size-at-outmigration of the returning (i.e. “successful”) adults from the same cohort (black bars). FLs given in 2mm bins (where the x-axis represents < that value, e.g. "55" = FL 53.01–55.0mm). Size classes used to categorize fry, parr and smolt outmigrants are indicated by dashed lines.

A strong, positive relationship was observed between OR and FL (*r* ^*2*^ = 0.92, n = 224, p < 0.001; FL = 0.171 (±0.003 SE) x OR—12.76 (±1.54 SE)), remaining linear across the full range of FLs reconstructed in the current study. This relationship was used to reconstruct FLs for individual ^87^Sr/^86^Sr profiles (e.g. Fig 3). The back-calculated size at which returning adults had outmigrated from the Stanislaus ranged from 31.3mm to 86.6mm in 2000, and 46.0mm to 90.5mm in 2003 (Fig 6). No yearlings were detected in the adult returns in either year.

To explore reproducibility of the method, paired left and right otoliths were analyzed from a subset of adults (n = 3 fry and n = 1 smolt outmigrant). All fish were assigned to the same migratory phenotype using either otolith, and the mean difference between back-calculated FLs was 2.3mm (e.g. Fig 3B).

### Contribution and survival of juvenile migratory phenotypes

The relative abundance of the migratory phenotypes in the escapement differed significantly to the outmigrating juvenile population in both 2000 (*X* ^2^ = 20,931, p<0.0001) and 2003 (*X* ^2^ = 1,381, p<0.0001). The phenotype composition of the adult population also differed significantly between years (*X* ^2^ = 749, *p*<0.0001), reflecting higher fry contributions in the wetter year (23% in 2000 *vs*.10% in 2003) and higher smolt contributions in the drier year (44% in 2003 *vs*. 13% in 2000). Despite representing only 10–16% of the outmigrating juveniles (Table 2), parr were the most commonly observed phenotype in the surviving adult populations (46–64%, Table 4), although parr and smolt contributions to the escapement were near-identical in 2003 (46% vs. 44%, respectively). Conversely, fry outmigrants represented 10–23% of the adult escapement, despite representing 50–85% of the juvenile sample (Tables 2 & 4). The lowest survival was observed in individuals <45mm, particularly in 2003, when the smallest outmigrant in the adult sample had left the river at 46mm FL, while the smallest individual captured in the RST was 27mm FL (Fig 6). Conversely, in 2000, 11% of the adults had left at FLs ≤46mm (the smallest at 31.3mm), compared with 80% of the original juvenile population (the smallest at 25mm; Fig 6).

**Table 4:** Contribution and survival of fry, parr and smolt outmigrants to the adult escapement.

Outmigration cohort	Phenotype	Contribution to the adult escapement (%) ^a^	No. spawners produced ^a^	Survival (%) ^b^
2000 (wetter)	Fry	23 (19–36)	1,334 (1112–2113)	0.07 (0.04–0.12)
	Parr	64 (43–66)	3,781 (2557–3892)	1.78 (1.15–2.76)
	Smolt	13 (9.4–25)	778 (556–1446)	0.77 (0.39–1.52)
2003 (drier)	Fry	10 (2.4–12)	148 (37–186)	0.19 (0.1–0.33)
	Parr	46 (34–61)	705 (520–928)	2.74 (1.73–4.34)
	Smolt	44 (34–59)	668 (520–891)	1.2 (0.78–1.87)

^a^ 95% CI in parentheses, derived from error around the FL back-calculation model.

^b^ 95% CI in parentheses, derived from error around the FL back-calculation and RST efficiency models

In both years, fry survival downstream of the Stanislaus (S_*fry*_) was significantly lower than parr or smolt survival (p<0.05). S_*parr*_ was approximately double S_*smolt*_ in both years, but the confidence intervals were overlapping (Table 4). Generally, outmigrant survival downstream of the Stanislaus was slightly higher in the drier year (2003) than the wetter year (2000), but significant differences were not detected (Table 4).

## Discussion

**Fig 7:**
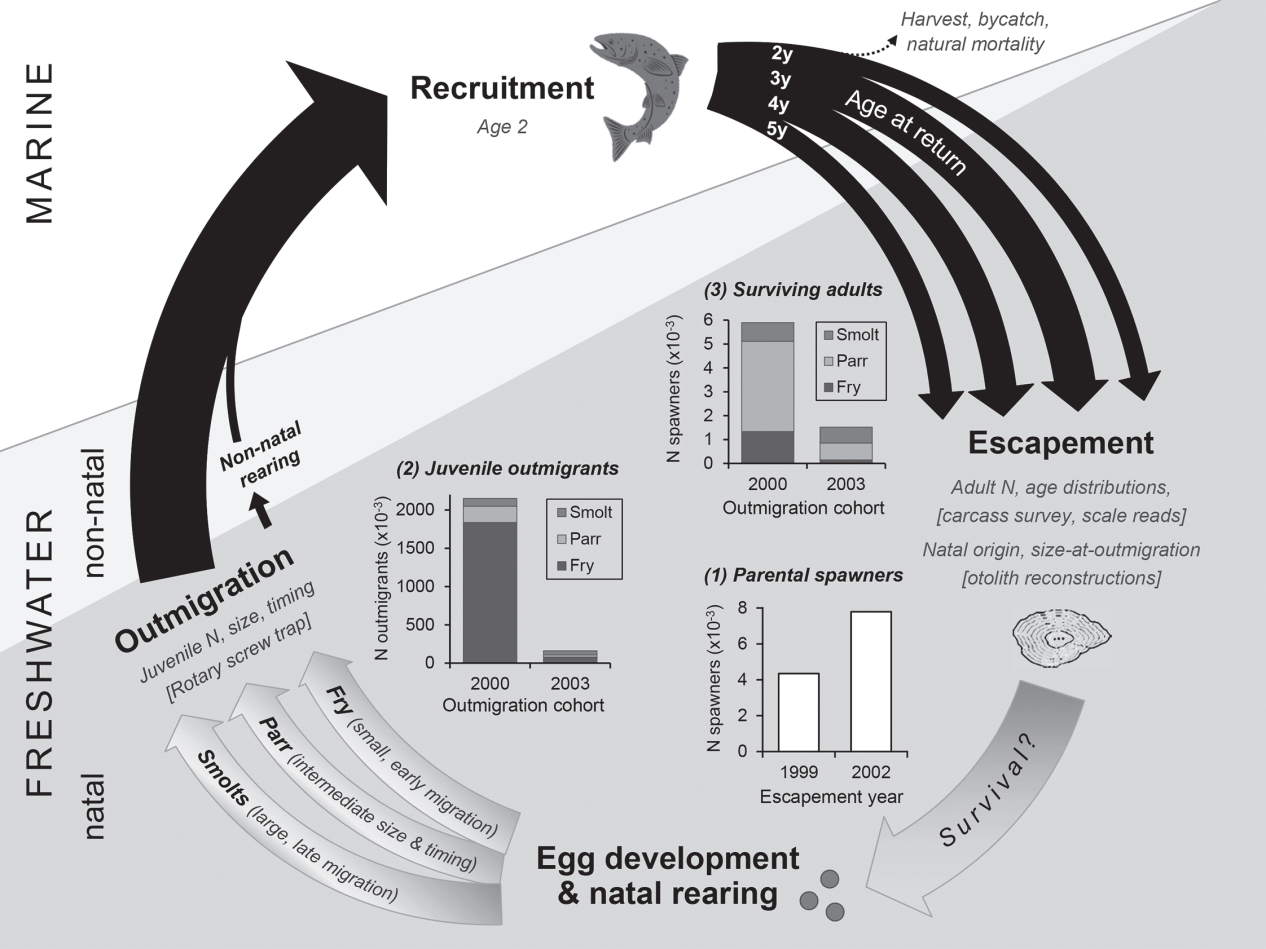
Schematic to conceptualize the data sources, methods and results presented in this study. This figure outlines the life cycle of fall-run Chinook salmon in the California Central Valley. Inset plot (1) demonstrates the abundance of parental spawners in the 1999 and 2002 escapement that contributed to the two focus years. Inset plots (2) and (3) illustrate the abundance and proportions of migratory phenotypes (fry, parr and smolts) observed in the juvenile sample (based on RST sampling) and in the adult escapement (based on otolith reconstructions), respectively. Arrow widths (not to scale) illustrate the typical proportions of 2, 3, 4 and 5 year olds observed in the adult escapement; note that age 5 fish tend to comprise <1% of the returns [50].

In this study we document the expression of juvenile salmon migratory phenotypes under two contrasting flow regimes and provide new insights into their contribution to the adult spawning population and ultimate survival. We observed variable expression and survivorship of fry, parr and smolt life histories within and between years, yet all three phenotypes consistently contributed to the adult spawning population. This result challenges the common perception in the CCV, that smolt outmigrants are the dominant phenotype driving adult population abundance. Our key findings in the context of the salmon life cycle in order to link the datasets, methods, and processes examined in the study (Fig 7). Overall, the wetter year (2000) was characterized by higher numbers of juvenile outmigrants and adult returns, despite fewer adult spawners contributing to the cohort the previous fall. Using the number of parental spawners as a coarse proxy for juvenile production, these trends suggest higher in-river mortality in the drier year (2003). Given similar downstream (outmigration-to-return) survival rates, these data suggest that for the two focus years of the study, cohort strength was primarily determined within the natal river, prior to juvenile outmigration.

### Juvenile outmigration behavior and phenotype expression

Juvenile outmigration timing in salmonids is inextricably linked to large-scale patterns in hydroclimatic regime and local-scale patterns in the magnitude, variation, and timing of flows [14,42]. In the Stanislaus, increases in flow were accompanied by pulses of outmigrants in both years, though greatly amplified during the turbid storm events of 2000. Correlations between fry migration, flow, and turbidity are commonly reported in the literature [14,53,54], and are suggested to have evolved as a result of reduced predation from visual piscivores [14,27,55,56]. The peak in migration in late January 2003 contained 85% of the year’s total fry outmigrants and coincided with a managed water release that resulted in mean river flows of 28.4 m^3^ s^-1^ [57]. This pulse flow appeared to stimulate fry migration, but comprised relatively clear water (~8 NTU) and contained outmigrants almost entirely <40mm FL (Fig 5). In both years, the larger parr- and smolt-sized fish also appeared to respond to instream flows, exhibiting smaller migration pulses from March through May, coincident with both natural and managed flows (Fig 4) [58,59].

The date and periods of peak migration were generally earlier and shorter in 2003, particularly for smolts. While warmer conditions can result in faster growth rates [60], smoltification in juvenile Chinook salmon is significantly impaired at temperatures above 15°C [61] and this critical temperature was reached at Ripon three weeks earlier in 2003, prior to the onset of peak parr migration. As the reduction in juvenile abundance in 2003 occurred in spite of greater numbers of parental spawners (Fig 7), we hypothesize that the truncation of migratory periods was driven by in-river mortality rather than altered migration timing or faster transitions between size classes. Juveniles tend to encounter less floodplain habitat, and increased predation rates and physiological stress in warmer, drier years [62], which likely resulted in a lower carrying capacity in the natal tributary [63] and increased density dependent mortality [64,65].

### Survival of migratory phenotypes

Although lower flows and warmer temperatures in the Stanislaus may have contributed to the lower outmigrant production observed in 2003, our results suggest that after exiting the natal river, there was no significant difference in juvenile survival. Survival rates were, if anything, marginally higher in 2003, contradicting many tagging studies which find reduced salmon survival through the freshwater delta during low flow conditions [24,66–68]. This discrepancy is likely due to differences in the sampling design and the time period represented by the different indices. Tagging studies generally release larger hatchery fish in similar sized batches during the later months of the outmigration season, when warmer conditions likely increase their vulnerability to predation [62]. Conversely, our survival estimates were based on variable numbers of fish over a larger size spectrum and broader migratory window, incorporating mortality events in all habitats downstream of the natal river, including the mainstem river, delta, estuary and ocean. However, we assume that differences in our survival indices would be driven by selective mortality events occurring during outmigration and early ocean residence. In support of this, there was no relationship between back-calculated size at outmigration and return FL (*r* ^2^ <0.01, *p*>0.05), implying that size-selective mortality did not vary by phenotype in the adult fish. However, marine distributions of adult salmon can be non-random [69], and if driven by timing at ocean entry, the migratory phenotypes could have been subjected to different ocean processes and mortality rates even as adults.

#### Parr and smolt outmigrants

Life history theory predicts selection to favor different phenotypes under different hydrologic regimes, maintaining behavioral and phenotypic diversity [70]. Yet in the current study, parr consistently exhibited the greatest contribution to the adult population and the highest survival rates. Greater representation of intermediary-sized juveniles has also been observed in some years in the ocean fisheries of Chinook [21] and Atlantic salmon [22], contradicting the expected directionality of size-selective mortality. Generally, larger or faster-growing individuals within a population are thought to have a selective advantage as a result of greater feeding opportunities, lower vulnerability to predation and greater tolerance of environmental perturbations [71]. However, the strength of size-selection in juvenile CCV Chinook salmon can vary as a function of ocean productivity [18], highlighting the importance of maintaining life history diversity in outmigration strategies. Without large-scale field experiments, it is not possible to definitively ascertain why smolts were not the most successful phenotype, however the San Joaquin basin is at the southernmost reaches of the species distribution [3] and its salmon populations are exposed to high temperatures, poor water quality, and significant water diversions [72,73]. This frequently results in river conditions that could impair growth and smoltification, and increased vulnerability to predation and disease [62], particularly at the end of the season when smolt-sized fish are most prevalent. Thus, the survival advantage of parr is likely attributable to both size and migration timing, analogous to the marine-orientated “critical size and period hypothesis” proposed by Beamish and Mahnken [74]. Furthermore, current flow practices in the San Joaquin basin include managed releases in April and May, intended to improve the survival of smolts [75]. These managed flows typically occur after most parr have left their natal tributaries, potentially selecting for this phenotype by providing downstream benefits as they migrate through (or rear in) the San Joaquin River and freshwater Delta.

#### Fry outmigrants

Little is known about the factors driving fry behavior or survival, yet the numbers that outmigrated during the wetter year (2000) were orders of magnitude higher, when they also contributed more than double the number of adult survivors (23% in 2000 *vs*. 10% in 2003). While fry consistently exhibited lower survival rates than their conspecifics (Table 4), reflecting the typical direction for size-selective mortality [71], the fact that any survived to contribute to the adult population, let alone contributing >20% of the adult returns, is a significant finding. Based on these data, their sheer abundance during high flow conditions at least partially helps to explain the increases in returns following wet outmigration conditions in the San Joaquin watershed (Fig 1). Early-migrating fry and parr may represent a significant portion of the population that can access favorable downstream rearing habitats in high flow years and survive to contribute to the adult population. Indeed, our otolith reconstructions indicated that all of the smallest (≤46mm FL) fry outmigrants in the surviving adult population (n = 4 in 2000, n = 1 in 2003) had spent several weeks rearing in the San Joaquin mainstem prior to leaving freshwater (e.g. Fig 3B). These data corroborate the extended transit times of CWT-tagged fish released in the San Joaquin basin and freshwater Delta in wetter years (averages of 16 d in 2000 vs. 6 d in 2003), although their mean size also differed (81mm vs. 87mm, respectively) [58]. Fry are observed in downstream freshwater and estuarine habitats in the CCV [20,76], and were probably more common when the Delta was a large tidal wetland [14,24,53]. This study confirms that these individuals can survive and contribute meaningfully to adult returns.

Currently there are no genetic data to support or refute a heritable component to early outmigration behavior, but it could otherwise meet the criteria of an adaptive trait, given that its expression is associated with “differential survival” and there is evidence for “a mechanism of selection” [77]. There is still some debate as to whether fry pulses during high flow events represent displacement due to reduced swimming ability or a deliberate behavior that might be considered a ‘strategy’ [3,14]. While catastrophic floods undoubtedly result in riverbed scouring and some fry displacement, not all individuals outmigrate during these events. Conversely, some fry migration is observed during periods with no pulse flows [78]. Given the frequency with which this phenotype is reported and the considerable rearing potential of downstream habitats, it is conceivable that fry dispersal is a heritable strategy, representing a ‘migratory contingent’ within the population [79,80]. Indeed, their consistent contribution to the adult population (observed here and in [21]) conclusively demonstrates that fry migration can be successful. If, however, early outmigration is purely an expression of phenotypic plasticity, it is likely that multiple factors are involved in stimulating the behavioral switch, including hydrology, intraspecific interactions [3] and density dependent mechanisms [65,81–83]. Irrespective of the underlying mechanisms, quantifying the relative success of migratory phenotypes across a broader range of hydrologic regimes is fundamental to understanding how environmental conditions and water operations contribute to salmon population dynamics.

### Otolith strontium isotopes and sources of uncertainty

One of the most significant advances of the current study was the pairing of RST sampling with otolith reconstructions. This process enabled us to compare fish size at a specific time and location across life stages, and provided a unique method for generating survival estimates into adulthood. CWT studies and acoustic telemetry have provided valuable insights into survival through particular stretches of the CCV [25,75], but tend to focus on larger fish and provide no information about the long-term success of particular traits. In addition, acoustic tags have focused on understanding flow-survival relationships for smolts, which are physiologically ready for seaward migration and likely use the mainstem rivers, delta, and estuary differently than fry or parr, which may exhibit prolonged rearing. Otolith ^87^Sr/^86^Sr ratios are an ideal natural tag as they vary among many of the rivers in the CCV, resulting in high classification scores for natal assignments (S1 and S2 Tables) [30,32,84]. Sr isotopes also represent a unique and sensitive marker for reconstructing downstream movements and non-natal rearing patterns in the freshwater system (e.g. Fig 3B). While seasonal variation in ^87^Sr/^86^Sr values have been reported in certain systems [85] and interannual variations were detected for some sites (S1 Table), these were minor compared with most of the geographic differences, with the majority of sites exhibiting classification scores >70% even when pooled across years (S2 Table). Importantly, the Stanislaus exhibited a stable and distinct isotopic signature; with 96% of juveniles correctly classified using jack-knife resampling (S2 Table). Identification of natal origin represents a significant advantage of using otolith Sr isotopes over element concentrations. This was critical for pairing RST- and otolith-derived datasets and providing confidence that our size reconstructions were not skewed by hatchery smolts.

A high occurrence of straying of fall-run Chinook salmon occurs between the San Joaquin and Sacramento basins [86–88], potentially due to the relative outflows during the return migration as well as hatchery release practices [89]. However the extent to which hatchery fish are functioning to sustain the San Joaquin salmon populations has gone largely undetected until recently [86,87]. In the current study, hatchery strays represented 18–51% of the unmarked fish, reducing the number of samples available to inform outmigration strategies of wild fish and increasing analytical costs. However, the removal of strays was vital to ensure that FL reconstructions were only performed on individuals that had experienced the same conditions as the RST-sampled juveniles. The implementation of 100% visual identification of hatchery fish [90] would increase the feasibility and efficiency of future life history diversity studies in this system.

We attempted to reduce and account for sources of uncertainty, but the low number of focus years and sample sizes, and the potential for error propagation limit the strength of our inferences. With greater representation of 2 and 4 year olds in our adult sample, a more sophisticated analysis using age-specific natal assignments could have been carried out. While no yearlings were detected in the surviving adults, their rarity in the RST-sampled outmigrant population indicate that larger sample sizes would be required to ascertain the success of this strategy with any confidence. Similarly, our approach for assigning natal origin based on otolith chemistry following yolk sac absorption means that individuals that outmigrated as yolk sac fry could have been misclassified as strays. However, yolk sac fry are rarely observed in the outmigrant population (0.1% of the 2001–2011 RST catch at Caswell), so this was deemed unlikely to significantly influence our results.

### Management implications

The complex biophysical properties of freshwater systems have led to the evolution of dynamic habitat mosaics [91] and diverse salmon life histories and distributions. The observed life history diversity likely provides within-population buffering, an as yet understudied component of the portfolio effect [5,6]. These data add to the mounting evidence that managing and conserving life history diversity is necessary to support resilient salmon populations, particularly in the face of climate change and projected human population growth [9,10]. Diversity in phenotypic traits is thought to produce a more stable population complex by decoupling population dynamics and buffering variance [6]. However, population resilience does not necessarily immediately translate into population abundance. In a highly regulated system such as the CCV, there is debate as to whether environmental unpredictability dictates a need to manage salmon stocks for diversity and resilience, or whether our understanding of (and control over) the relevant processes is sufficient to manage purely for abundance. Such topics are complicated by socio-economic and ecological trade-offs, however, by improving our understanding of how juvenile life history strategies are expressed and respond to different flow regimes, we may be able to optimize both. Currently, the portfolio effect for CCV salmon stocks is weak and deteriorating [92] and San Joaquin populations face serious future challenges, given predicted 25–40% reductions in snowmelt by 2050 [93]. CCV salmon exhibit diverse outmigration timings that have evolved over geological time scales in response to the unpredictable hydroclimatic conditions characteristic of the region [11]. Yet modern-day water and hatchery management practices tend to constrain outmigration timing. For example, alterations to the natural hydrograph, such as suppression of winter pulse flows, likely to truncate migratory windows, reduce the variability in outmigration timing, and significantly suppress the fry life history type. Such simplification and truncation of life history diversity could significantly reduce the resiliency of the stock-complex and exacerbate the risk of a temporal mismatch with favorable ocean conditions [94]. Indeed, the only clear deviation from the flow-driven relationship in Fig 1 was attributed to juveniles entering the ocean during a suboptimal period and resulted in the closure of the fishery in 2008. Perhaps with more diverse, resilient stocks, the consequences would have been less extreme. Largely without direct empirical support, hatchery and flow management practices tend to focus on optimizing the success of the largest, smolt-sized juveniles that are assumed to contribute the most to adult returns [14,21,24]. Here, we found that all phenotypes contributed to the reproductive adult population, with smolts comprising less than half of the surviving adults following two contrasting flow regimes. Without otolith reconstruction data for additional years, species, and watersheds, the broader inferences one can make regarding the influence of hydroclimatic regime on juvenile salmon survival are limited. However our data and a previous study [21] indicate that assumptions regarding size-selective mortality and smolt-focused management schemes need to be tested on a species, system and hydroclimatic basis.

This study has demonstrated the value of a combined RST and otolith geochemistry study to reconstruct patterns in the expression and survival of salmon migratory phenotypes. The results show that under paired years of low and high flow conditions, parr outmigrants comprised a significant portion of the returning adult population, while fry made smaller, but substantial contributions. Future efforts should focus on reducing the error in juvenile production estimates in order to produce more meaningful survival estimates, and understanding the demographic role that fry and parr play in salmon population dynamics. Management actions that promoted the expression and survival of fry in natal and downstream rearing habitats could result in demographic and genetic benefits to the population. Recognition of the importance of hydrodynamic regime and life history diversity should provide guidance to system managers when reassessing goals and future management strategies [5,95]. It is also important that management actions consider carefully-designed monitoring programs to detect changes in stock abundance and life history diversity at appropriate temporal and spatial scales.

## Supporting Information

S1 Text Testing the performance of the Sr isoscape. (DOCX)

S1 Fig Time-resolved plot of a single spot ablation at a habitat transition. This plot (macro developed by C. Donohoe) shows how the isotopic composition of the otolith can change with sample depth (equivalent to analysis time). Typically we would use ~20 seconds of data per spot (A), but in cases like this we would use only the surface material (B) to avoid signal attenuation and to ensure consistency between otolith ^87^Sr/^86^Sr, microstructure and distance analyses.
(DOCX)

S2 Fig Median ^87^Sr/^86^Sr natal values for major sources of Chinook salmon in the California Central Valley. Values are based on juvenile otoliths and/or water samples. The mainstem San Joaquin River (SJR) isotopic signature is displayed, but was not included as a potential natal source. Boxes represent 25-75^th^ percentiles, whiskers represent 5-95^th^ percentiles. Site codes are defined in S1 Table. Isotopic signatures not significantly different (*p* > 0.05, Tukey’s test) are joined by brackets. Mean ocean ^87^Sr/^86^Sr is indicated by a dashed line.
(TIF)

S1 Table ^87^Sr/^86^Sr isoscape used to train the LDFA and assign unknown adult otoliths to natal location. Data based on known-origin otolith (O) and/or water (W) samples. Interannual differences were tested by ANOVA or Welch's Test when data exhibited unequal variance. Differences among sites are shown in S2 Fig Underlined years represent water samples collected Oct 1997 to Apr 1998 that were pooled into a single water year (1998).
(DOCX)

S2 Table Natal assignments and correct classification scores of known-origin samples. Assignments based on ^87^Sr/^86^Sr values and jackknife resampling. Site codes are defined in S1 Table. Equal prior probabilities were given to all sites and sites are ordered by increasing mean ^87^Sr/^86^Sr value. The training dataset (n = 290) comprised both juvenile otoliths and water samples. Counts are for actual rows by predicted columns. Samples from the Stanislaus River (STA) are highlighted in bold, while groups of sites with statistically overlapping ^87^Sr/^86^Sr signatures (*p*>0.05, Tukey’s test) are shown in italics and S2 Fig
(DOCX)

S3 Table Reference samples used to calibrate the fork length back-calculation model. (DOCX)

S4 Table The number of adult spawners produced by the 2000 and 2003 outmigration cohorts (“natural escapement”) (DOCX)
