# Supplementary material for: Reconstructing the Migratory Behavior and Long-Term Survivorship of Juvenile Chinook Salmon under Contrasting Hydrologic Regimes
Source: PLoS One. 2015 May 20;10(5):e0122380. doi: 10.1371/journal.pone.0122380 (PMC4439044; doi:10.1371/journal.pone.0122380)
Supplement: S1 Text — (DOCX) [file pone.0122380.s001.docx]

# Supporting Information

# S1 Text. Testing the performance of the Sr isoscape

The ^87^Sr/^86^Sr signature of otolith material deposited immediately following onset of exogenous feeding (~250µm from the core, after [[1](#_ENREF_1)]) was characterized in juveniles collected from their natal tributary or hatchery. The ^87^Sr/^86^Sr baseline was further augmented through inclusion of water samples from non-hatchery sites [[2](#_ENREF_2)] and the San Joaquin River (P. Weber unpublished). Differences in ^87^Sr/^86^Sr signatures among years and sites were tested by ANOVA (or Welch’s Test if data violated assumptions of equal variance, JMP 9.0) and Tukey’s *post hoc* pairwise comparisons (S1 Fig. and S1 Table). The accuracy of the LDFA model used to predict natal origin of adult fish was evaluated using the proportion of reference samples correctly classified using jackknife resampling (MYSTAT 12.02) (S2 Table).

Natal ^87^Sr/^86^Sr values differed significantly among sites (F_16, 61.73_ = 10 118, p<0.001 Welch’s test, JMP 9.0). Eight sites, including the Stanislaus, were fully unique (*p*<0.05, S1 Table and S2 Fig.). The Stanislaus exhibited a stable and distinct isotopic signature, with 96% of Stanislaus-origin juveniles correctly classified (S2 Table). Of the 27 CWT-tagged adults included blind in the LDFA assignments, none were incorrectly classified to the Stanislaus. All Merced- (n=14) and 85% of Mokelumne-origin (n=11 of 13) fish were correctly assigned to their hatchery source. The two incorrectly classified Mokelumne Hatchery fish were assigned to the Tuolumne River, and the isoscape confirmed the existence of overlap between these two sources (S1 and S2 Tables). Given lower posterior probability of Tuolumne River assignments (all <0.6) and typically limited straying among wild populations [usually <5%, 56], all Tuolumne-assigned fish were *post hoc* reclassified to the Mokelumne Hatchery (n = 3 from 2000, including the two CWT fish; n = 7 from 2003).

# References

1. Barnett-Johnson R, Ramos FC, Grimes CB, MacFarlane RB (2005) Validation of Sr isotopes in otoliths by laser ablation multicollector inductively coupled plasma mass spectrometry (LA-MC-ICPMS): opening avenues in fisheries science applications. Canadian Journal of Fisheries and Aquatic Sciences 62: 2425-2430.

2. Ingram LB, Weber PK (1999) Salmon origin in California's Sacramento–San Joaquin river system as determined by otolith strontium isotopic composition. Geology 27: 851-854.
