# Supplementary material for: Reconstructing the Migratory Behavior and Long-Term Survivorship of Juvenile Chinook Salmon under Contrasting Hydrologic Regimes
Source: PLoS One. 2015 May 20;10(5):e0122380. doi: 10.1371/journal.pone.0122380 (PMC4439044; doi:10.1371/journal.pone.0122380)
Supplement: S1 Fig — This plot (macro developed by C. Donohoe) shows how the isotopic composition of the otolith can change with sample depth (equivalent to analysis time). Typically we would use ~20 seconds of data per spot (A), but in cases like this we would use only the surface material (B) to avoid signal attenuation and to ensure consistency between otolith 87Sr/86Sr, microstructure and distance analyses. (DOCX) [file pone.0122380.s002.docx]

**S1 Fig. Time-resolved plot of a single spot ablation at a habitat transition.** This plot (macro developed by C. Donohoe) shows how the isotopic composition of the otolith can change with sample depth (equivalent to analysis time). Typically we would use ~20 seconds of data per spot (A), but in cases like this we would use only the surface material (B) to avoid signal attenuation and to ensure consistency between otolith ^87^Sr/^86^Sr, microstructure and distance analyses.

**
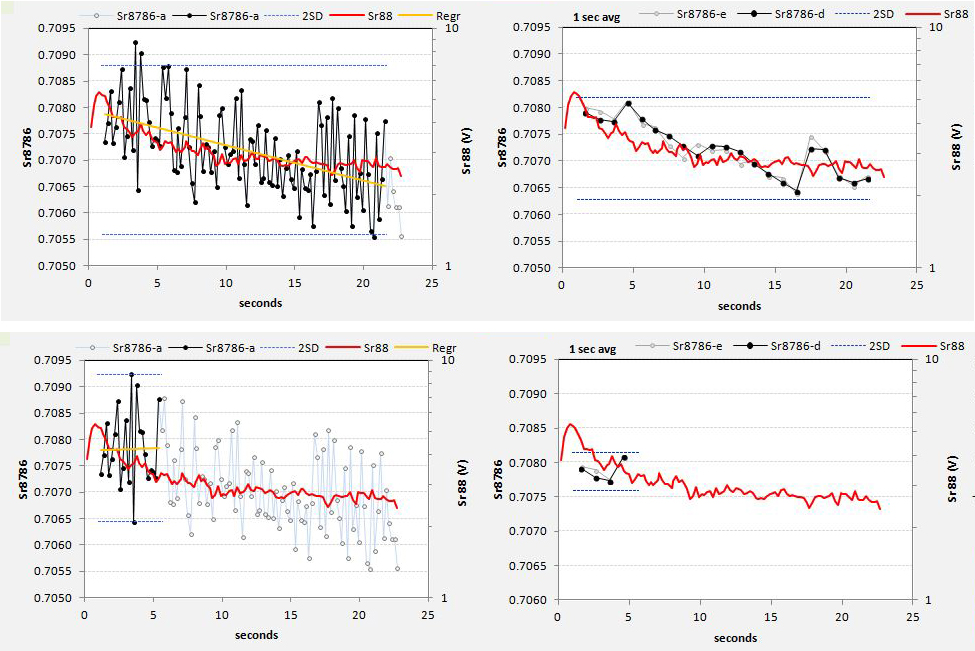
**

**A**

**B**
