# Supplementary figures and images for: Reconstructing the Migratory Behavior and Long-Term Survivorship of Juvenile Chinook Salmon under Contrasting Hydrologic Regimes
Source: PLoS One. 2015 May 20;10(5):e0122380. doi: 10.1371/journal.pone.0122380 (PMC4439044; doi:10.1371/journal.pone.0122380)

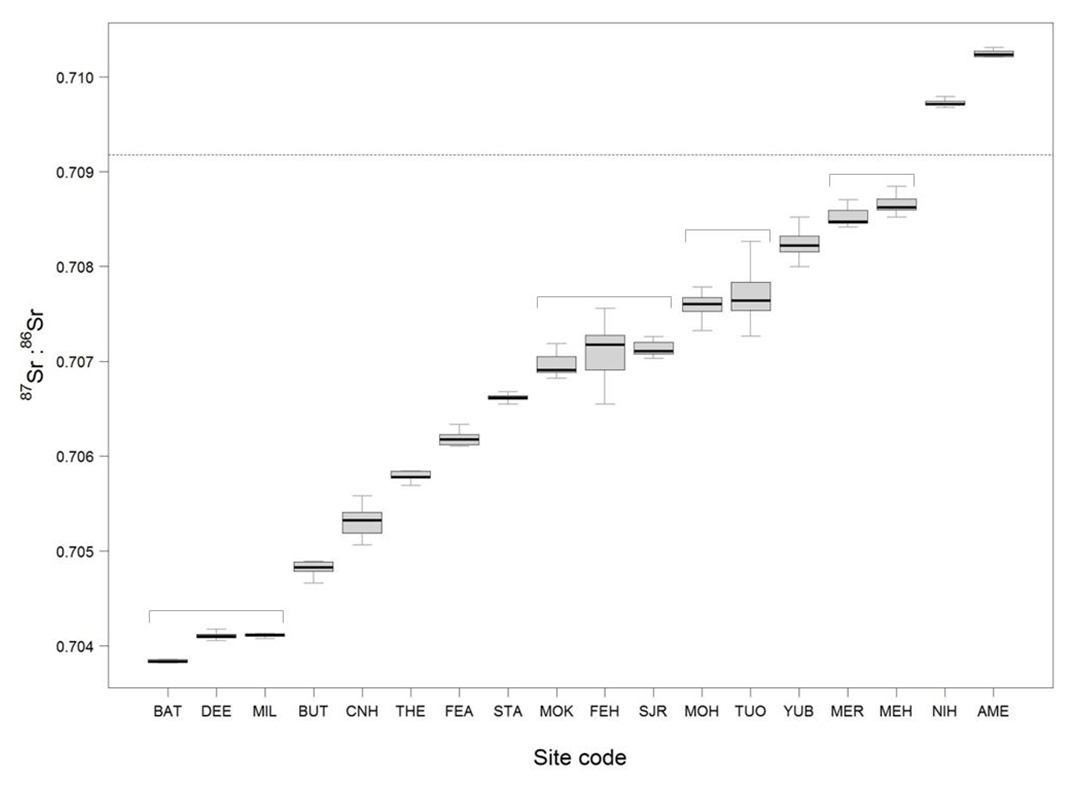

Supplement: S2 Fig — Values are based on juvenile otoliths and/or water samples. The mainstem San Joaquin River (SJR) isotopic signature is displayed, but was not included as a potential natal source. Boxes represent 25-75th percentiles, whiskers represent 5-95th percentiles. Site codes are defined in S1 Table. Isotopic signatures not significantly different (p > 0.05, Tukey’s test) are joined by brackets. Mean ocean 87Sr/86Sr is indicated by a dashed line. (TIF) [file pone.0122380.s003.tif]
