# Supplementary material for: Reconstructing the Migratory Behavior and Long-Term Survivorship of Juvenile Chinook Salmon under Contrasting Hydrologic Regimes
Source: PLoS One. 2015 May 20;10(5):e0122380. doi: 10.1371/journal.pone.0122380 (PMC4439044; doi:10.1371/journal.pone.0122380)
Supplement: S1 Table — Data based on known-origin otolith (O) and/or water (W) samples. Interannual differences were tested by ANOVA or Welch's Test when data exhibited unequal variance. Differences among sites are shown in S2 Fig Underlined years represent water samples collected Oct 1997 to Apr 1998 that were pooled into a single water year (1998). (DOCX) [file pone.0122380.s004.docx]

#### **S1 Table.** **^87^Sr/^86^Sr isoscape used to train the LDFA and assign unknown adult otoliths to natal location.**

| **Site** | **Site code** | **Source** | **Sample year(s)** | **N** | **Sample type** | **Average ^87^Sr/^86^Sr (± SD)** | | **Interannual difference** |
| --- | --- | --- | --- | --- | --- | --- | --- | --- |
| American River | AME | [[1](#_ENREF_1)] | 1999 | 5 | O | 0.71025 | (0.00004) | n/a ^a^ |
| Nimbus Hatchery | NIH | [[1](#_ENREF_1)] | 2002 | 9 | O | 0.70974 | (0.00006) | n/a |
| Merced Hatchery | MEH | [[1](#_ENREF_1)], Johnson et al. (unpubl.) | 1999, 2002, 2004 | 15 | O | 0.70865 | (0.00009) | N.S. |
| Merced River | MER | [[1](#_ENREF_1)] | 2003 | 13 | O | 0.70852 | (0.00010) | n/a |
| Yuba River | YUB | [[1](#_ENREF_1)], Johnson et al. (unpubl.) | 2002 | 19 | O | 0.70823 | (0.00021) | n/a |
| Tuolumne River | TUO | [[1](#_ENREF_1),[2](#_ENREF_2)], Johnson et al. (unpubl.) | 1997, 1998, 1999, 2003, 2007, 2010 | 55 | O | 0.70768 | (0.00022) | F_4, 50_ = 3.08, p = 0.024  (no pairwise differences) |
| Mokelumne Hatchery | MOH | [[1](#_ENREF_1)], Johnson et al. (unpubl.) | 1999, 2000, 2002 | 20 | O | 0.70759 | (0.00012) | N.S. |
| San Joaquin River | SJR | [[2](#_ENREF_2)], Weber (unpubl.) | 1998, 1999, 2000 | 7 | W | 0.70716 | (0.00013) | N.S. |
| Feather Hatchery | FEH | [[1](#_ENREF_1)], Johnson et al. (unpubl.) | 1999, 2000, 2002, 2004 | 32 | O | 0.70712 | (0.00025) | F_3, 28_ = 11.4, p < 0.0001  (1999 < 2000, 2002, 2004) |
| Mokelumne River | MOK | [[1](#_ENREF_1),[2](#_ENREF_2)], Johnson et al. (unpubl.) | 1997, 1998, 2000, 2002 | 18 | W, O | 0.70695 | (0.00011) | F_2, 4.9_ = 22.7, p = 0.003 (Welch's Test); 2000 > 1998, 2002 |
| Stanislaus River | STA | [[1](#_ENREF_1)], Johnson et al. (unpubl.) | 1999, 2000, 2002 | 24 | O | 0.70660 | (0.00008) | N.S. |
| Feather River | FEA | [[1](#_ENREF_1),[2](#_ENREF_2)], Johnson et al. (unpubl.) | 1997, 1998, 2000, 2002 | 25 | W, O | 0.70619 | (0.00012) | N.S. |
| Thermalito Annex | THE | [[1](#_ENREF_1)] | 2004 | 5 | O | 0.70581 | (0.00011) | n/a |
| Coleman Hatchery | CNH | [[1](#_ENREF_1)] | 2000, 2002 | 13 | O | 0.70533 | (0.00031) | N.S. |
| Butte Creek | BUT | [[2](#_ENREF_2)] | 1997, 1998 | 5 | W | 0.70481 | (0.00009) | n/a |
| Mill Creek | MIL | [[1](#_ENREF_1)] | 2002 | 10 | O | 0.70412 | (0.00003) | n/a ^a^ |
| Deer Creek | DEE | [[1](#_ENREF_1),[2](#_ENREF_2)] | 1997, 1998, 2002 | 13 | W, O | 0.70410 | (0.00003) | N.S. |
| Battle Creek | BAT | [[1](#_ENREF_1)] | 1999 | 9 | O | 0.70390 | (0.00014) | n/a |

#### Data based on known-origin otolith (O) and/or water (W) samples. Interannual differences were tested by ANOVA or Welch's Test when data exhibited unequal variance. Differences among sites are shown in S2 Fig. Underlined years represent water samples collected Oct 1997 to Apr 1998 [[2](#_ENREF_2)] that were pooled into a single water year (1998).

^a^ A significant difference was observed between otoliths and water samples collected in two different years. However, as only two years were available, it was not possible to distinguish interannual differences from matrix-related differences, so for these sites, the water samples were excluded from the isotopic baseline.

1. Barnett-Johnson R, Pearson T, Ramos F, Grimes C, MacFarlane R (2008) Tracking natal origins of salmon using isotopes, otoliths, and landscape geology. Limnology and Oceanography 53: 1633-1642.

2. Ingram LB, Weber PK (1999) Salmon origin in California's Sacramento–San Joaquin river system as determined by otolith strontium isotopic composition. Geology 27: 851-854.
