# Supplementary material for: Reconstructing the Migratory Behavior and Long-Term Survivorship of Juvenile Chinook Salmon under Contrasting Hydrologic Regimes
Source: PLoS One. 2015 May 20;10(5):e0122380. doi: 10.1371/journal.pone.0122380 (PMC4439044; doi:10.1371/journal.pone.0122380)
Supplement: S2 Table — Assignments based on 87Sr/86Sr values and jackknife resampling. Site codes are defined in S1 Table. Equal prior probabilities were given to all sites and sites are ordered by increasing mean 87Sr/86Sr value. The training dataset (n = 290) comprised both juvenile otoliths and water samples. Counts are for actual rows by predicted columns. Samples from the Stanislaus River (STA) are highlighted in bold, while groups of sites with statistically overlapping 87Sr/86Sr signatures (p>0.05, Tukey’s test) are shown in italics and S2 Fig (DOCX) [file pone.0122380.s005.docx]

#### **S2 Table. Natal assignments and correct classification scores of known-origin samples.**

|  | BAT | DEE | MIL | BUT | CNH | THE | FEA | STA | MOK | FEH | MOH | TUO | YUB | MER | MEH | NIH | AME | % correct |
| --- | --- | --- | --- | --- | --- | --- | --- | --- | --- | --- | --- | --- | --- | --- | --- | --- | --- | --- |
| BAT | *7* | *1* | *1* | 0 | 0 | 0 | 0 | 0 | 0 | 0 | 0 | 0 | 0 | 0 | 0 | 0 | 0 | 78 |
| DEE | *0* | *8* | *5* | 0 | 0 | 0 | 0 | 0 | 0 | 0 | 0 | 0 | 0 | 0 | 0 | 0 | 0 | 62 |
| MIL | *0* | *3* | *7* | 0 | 0 | 0 | 0 | 0 | 0 | 0 | 0 | 0 | 0 | 0 | 0 | 0 | 0 | 70 |
| BUT | 0 | 0 | 0 | 5 | 0 | 0 | 0 | 0 | 0 | 0 | 0 | 0 | 0 | 0 | 0 | 0 | 0 | 100 |
| CNH | 0 | 0 | 0 | 2 | 9 | 1 | 1 | 0 | 0 | 0 | 0 | 0 | 0 | 0 | 0 | 0 | 0 | 69 |
| THE | 0 | 0 | 0 | 0 | 0 | 4 | 1 | 0 | 0 | 0 | 0 | 0 | 0 | 0 | 0 | 0 | 0 | 80 |
| FEA | 0 | 0 | 0 | 0 | 0 | 1 | 22 | 2 | 0 | 0 | 0 | 0 | 0 | 0 | 0 | 0 | 0 | 88 |
| **STA** | **0** | **0** | **0** | **0** | **0** | **0** | **1** | **23** | **0** | **0** | **0** | **0** | **0** | **0** | **0** | **0** | **0** | 96 |
| MOK | 0 | 0 | 0 | 0 | 0 | 0 | 0 | 0 | *13* | *5* | 0 | 0 | 0 | 0 | 0 | 0 | 0 | 72 |
| FEH | 0 | 0 | 0 | 0 | 0 | 0 | 0 | 3 | *8* | *14* | 7 | 0 | 0 | 0 | 0 | 0 | 0 | 44 |
| MOH | 0 | 0 | 0 | 0 | 0 | 0 | 0 | 0 | 0 | 1 | *11* | *8* | 0 | 0 | 0 | 0 | 0 | 55 |
| TUO | 0 | 0 | 0 | 0 | 0 | 0 | 0 | 0 | 0 | 3 | *22* | *25* | 5 | 0 | 0 | 0 | 0 | 45 |
| YUB | 0 | 0 | 0 | 0 | 0 | 0 | 0 | 0 | 0 | 0 | 1 | 0 | 14 | 3 | 1 | 0 | 0 | 74 |
| MER | 0 | 0 | 0 | 0 | 0 | 0 | 0 | 0 | 0 | 0 | 0 | 0 | 0 | *9* | *4* | 0 | 0 | 69 |
| MEH | 0 | 0 | 0 | 0 | 0 | 0 | 0 | 0 | 0 | 0 | 0 | 0 | 0 | *3* | *12* | 0 | 0 | 80 |
| NIH | 0 | 0 | 0 | 0 | 0 | 0 | 0 | 0 | 0 | 0 | 0 | 0 | 0 | 0 | 0 | 9 | 0 | 100 |
| AME | 0 | 0 | 0 | 0 | 0 | 0 | 0 | 0 | 0 | 0 | 0 | 0 | 0 | 0 | 0 | 0 | 5 | 100 |
| Total | 7 | 12 | 13 | 7 | 9 | 6 | 25 | 28 | 21 | 23 | 41 | 33 | 19 | 15 | 17 | 9 | 5 | 68 |

#### Assignments based on ^87^Sr/^86^Sr values and jackknife resampling. Site codes are defined in S1 Table. Equal prior probabilities were given to all sites and sites are ordered by increasing mean ^87^Sr/^86^Sr value. The training dataset (n = 290) comprised both juvenile otoliths and water samples. Counts are for actual rows by predicted columns. Samples from the Stanislaus River (STA) are highlighted in bold, while groups of sites with statistically overlapping ^87^Sr/^86^Sr signatures (*p*>0.05, Tukey’s test) are shown in italics and S2 Fig.
