# Supplementary material for: Reconstructing the Migratory Behavior and Long-Term Survivorship of Juvenile Chinook Salmon under Contrasting Hydrologic Regimes
Source: PLoS One. 2015 May 20;10(5):e0122380. doi: 10.1371/journal.pone.0122380 (PMC4439044; doi:10.1371/journal.pone.0122380)
Supplement: S3 Table — (DOCX) [file pone.0122380.s006.docx]

#### **S3 Table. Reference samples used to calibrate the fork length back-calculation model.**

| **Collection site** | **N** | **Year(s)** | **Mean fork length [range] (mm)** |
| --- | --- | --- | --- |
| Coleman Hatchery | 40 | 2002 | 33.0 [28.2 - 37.5] |
| Golden Gate Bridge | 83 | 2005 | 83.6 [62 – 132] |
| Stanislaus River | 95 | 2000, 2002, 2013 | 59.1 [27.5 – 94] |
| Tuolumne River | 6 | 2003 | 79.3 [71 – 87] |
| Total | 224 |  |  |
