# Supplementary material for: Reconstructing the Migratory Behavior and Long-Term Survivorship of Juvenile Chinook Salmon under Contrasting Hydrologic Regimes
Source: PLoS One. 2015 May 20;10(5):e0122380. doi: 10.1371/journal.pone.0122380 (PMC4439044; doi:10.1371/journal.pone.0122380)
Supplement: S4 Table — (DOCX) [file pone.0122380.s007.docx]

#### **S4 Table.** **The number of adult spawners produced by the 2000 and 2003 outmigration cohorts (“natural escapement”)**

|  | | | | | | | | Age distribution ^c, d^ | | | |  | | |
| --- | --- | --- | --- | --- | --- | --- | --- | --- | --- | --- | --- | --- | --- | --- |
| Cohort | Escapement year | Total escapement  a | N adclipped  fish sampled  ^b^ | Tag recovery rate ^c^ | Estimated total N adclipped fish | Percent adclipped | N unmarked | Age 2  (%) | Age 3  (%) | Age 4  (%) | Age 5  (%) | N unmarked  by cohort | Percent unmarked hatchery strays (Table 3) | Natural escapement by cohort |
| 2000 | 2001 | 7,033 | 41 | 0.157 | 261 | 4 | 6,772 | **13** | 33 | 54 | 0.38 | 7,228 | 18 | 5893 |
|  | 2002 | 7,787 | 201 | 0.637 | 316 | 4 | 7,471 | 15 | **62** | 23 | 0.71 |  |  |  |
|  | 2003 | 5,902 | 235 | 0.321 | 732 | 12 | 5,170 | 13 | 53 | **33** | 0.23 |  |  |  |
| 2003 | 2004 | 4,015 | 86 | 0.528 | 163 | 4 | 3,852 | **30** | 44 | 25 | **0.30** | 3,081 | 51 | 1522 |
|  | 2005 | 1,427 | 18 | 0.415 | 43 | 3 | 1,384 | 7 | **77** | 16 | 0.18 |  |  |  |
|  | 2006 | 1,923 | 9 | 0.335 | 27 | 1 | 1,896 | 26 | 29 | **45** | 0.18 |  |  |  |
|  | 2007 | 443 | 0 | 0 | 0 | 0 | 443 | 12 | 76 | 12 | **0.36** |  |  |  |

^a^ CDFW GrandTab (www.calfish.org)

^b^ Regional Mark Information System (www.rmis.org). Query: “All recoveries”, Recovery location code: “6FCSJSTA”, Recorded Mark: “Adclip”, Years: 2001-2007

^c^ Mesick C, Marston D, Heyne T (2009) Estimating recruitment for fall-run Chinook salmon populations in the Stanislaus, Tuolumne, and Merced Rivers. Instream Energy Flow Branch USFWS. (http://www.waterboards.ca.gov/waterrights/water_issues/programs/bay_delta/deltaflow/cspa.shtml)

^d^ Individuals that outmigrated in 2000 and 2003 are highlighted in bold
